# Anion gap associated with 28-days all-cause mortality in Acute cholangitis patients admitted to the intensive care unit in MIMIC-IV database: a retrospective cohort study

**DOI:** 10.3389/fmed.2025.1591096

**Published:** 2025-05-23

**Authors:** Yingjie Huang, Zhijie Yin, Wei Han

**Affiliations:** Department of Pancreatic Surgery, The First Affiliated Hospital of Xinjiang Medical University, Ürümqi, China

**Keywords:** anion gap (AG), acute cholangitis, 28-days all-cause mortality, intensive care unit (ICU), MIMIC-IV database

## Abstract

**Background:**

Acute cholangitis, characterized by infection of the bile duct, represents a significant clinical challenge due to its association with heightened morbidity and mortality rates. This condition often culminates in severe complications, including sepsis and multi-organ failure, ultimately leading to increased healthcare burdens. The anion gap (AG) serves as a potential biomarker for systemic inflammation and has been proposed as a prognostic indicator. To evaluate its efficacy in predicting patient outcomes, a closer examination of AG levels and their relationship to mortality in acute cholangitis patients is warranted.

**Methods:**

This study employed a retrospective cohort design, utilizing data gleaned from the MIMIC-IV database. A total of 489 patients admitted to the Intensive Care Unit (ICU) with acute cholangitis were analyzed, and participants were stratified into quartiles according to their serum AG levels. Mortality rates, as well as the incidence of acute kidney injury (AKI) and sepsis, were meticulously recorded and analyzed to establish any significant correlations with AG levels.

**Results:**

The findings indicated a stark association between elevated AG quartiles and increased rates of AKI, sepsis, and overall mortality. Specifically, the 28-day mortality rate escalated markedly from 8.1% in the lowest AG quartile to 30.9% in the highest quartile (*p* < 0.001). Furthermore, multivariate logistic regression analysis revealed that each unit increase in AG was associated with a 13% enhancement in mortality risk (OR 1.13, 95%CI 1.03–1.124, *p* = 0.010). An inverted J-shaped correlation between AG levels and mortality was also identified, indicating a potential inflection point at 18.13 mEq/L.

**Conclusion:**

This study elucidates the significant role of AG as a prognostic marker in critically ill patients with acute cholangitis, emphasizing its potential utility in guiding early intervention strategies to mitigate mortality risks. Future research endeavors should aim to explore the therapeutic implications of managing AG levels and assess their relevance in wider clinical contexts to enhance patient outcomes.

## Introduction

Acute cholangitis is a serious infection of the bile duct characterized by the classical triad of fever, jaundice, and abdominal pain (1). It often results from biliary obstruction, which may arise from conditions such as choledocholithiasis, strictures, or malignancies (2, 3). The disease’s acute nature can lead to rapid clinical deterioration, which poses significant challenges for healthcare systems due to the high mortality rates associated with severe cases (4). Health professionals must recognize the importance of early diagnosis and appropriate management to mitigate the risk of complications, including organ dysfunction and septic shock, which can arise from the systemic inflammatory response syndrome (SIRS) associated with (5).

One potential biomarker identified in recent studies is the serum anion gap (AG), which has been associated with systemic inflammation and organ failure in critically ill patients. The AG provides insight into the metabolic derangements and can reflect the severity of illness (6, 7). Previous studies have indicated that elevated AG levels correlate with worse outcomes in various clinical settings, including sepsis and acute respiratory distress syndrome (8–10). Thus, examining the relationship between AG levels and mortality in acute cholangitis patients could provide valuable information for clinicians aiming to optimize patient management strategies.

While AG has been studied in sepsis and ARDS, its prognostic role in acute cholangitis remains underexplored, making this study a novel contribution to hepatobiliary critical care. By establishing a correlation between AG levels and clinical outcomes, this study seeks to explore promising applications regarding this prognostic marker for critical illness. The findings from this investigation have the potential to influence clinical practice by providing insights that can lead to more tailored approaches to managing acute cholangitis and improving patient survival rates.

## Materials and methods

We meticulously assembled a retrospective cohort from the MIMIC-IV database, aiming to explore the correlation between AG and the 28-day all-cause mortality rate in patients with acute cholangitis. The MIMIC-IV database contains comprehensive clinical data on patients admitted to the ICU at Beth Israel Deaconess Medical Center (BIDMC) between 2008 and 2022. Researchers can access the MIMIC-IV database after completing a human research participant protection training course and signing a Data Use Agreement (DUA). The Data Use Agreement requires users to adequately protect the dataset by not attempting to re-identify individuals and not sharing data. It also requires reporting of any issues related to de-identification (11). In response to the requirement, Yingjie Huang completed the online NIH course on ‘Protecting Human Research Participants’, successfully passed the corresponding examination, and subsequently gained authorized access to the database (Record ID: 13445533).

### Study population

**FIGURE 1:**
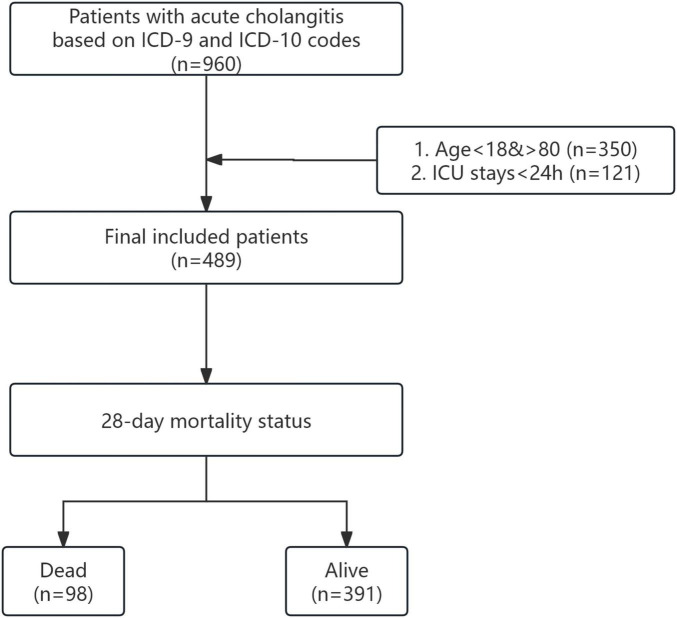
Flowchart of participant selection.

Our research included adults with acute cholangitis, aged 18 to 80, who were initially admitted to the intensive care unit and had a hospital stay exceeding 24 h. Acute cholangitis patients were identified through ICD-9 code 5761 and ICD-10 code K830. Our exclusion criteria were patients who stayed in the intensive care unit for less than 24 h. To ensure data quality and reliability, we excluded patients with a missing data rate exceeding 20% and focusing on patients with relatively complete records for more accurate and reliable analysis. The corresponding flowchart illustrating the study’s inclusion and exclusion criteria is presented in Figure 1.

### Clinical variables

Utilizing PgAdmin4, we executed structured query language (SQL) to extract data from the MIMIC IV database. Data extraction focused on six principal categories: (1) demographic characteristics, including age, gender, and race; (2) comorbid conditions, such as hypertension, diabetes; (3) laboratory indicators, comprising red blood cell count (RBC), white blood cell count (WBC), red cell distribution width (RDW), hemoglobin, hematocrit, anion gap, sodium, Potassium, platelet count (PLT), prothrombin time (PT), partial thromboplastin time (PTT), international normalized ratio (INR), alanine aminotransferase (ALT), aspartate aminotransferase (AST), bilirubin total, urea nitrogen and creatinine; (4) treatments, continuous renal replacement therapy (CRRT); (5) severity of illness scores, namely Simplified Acute Physiology Score II (SAPS II) and Sequential Organ Failure Assessment (SOFA) (12, 13).

### Research variable and outcomes

The primary variable in this study was the anion gap (AG), defined as the first recorded AG within 24 h of ICU admission. The main outcome measured was the 28-day all-cause mortality in patients with acute cholangitis (14).

### Statistical analysis

Descriptive analysis was performed on the entire patient sample. Normality was examined using the Shapiro-Wilk test. Continuous variables exhibiting normal distribution were expressed as mean with standard deviation (SD); those with skewed distribution were detailed as median and interquartile range (IQR). Categorical variables were depicted in terms of frequency counts and percentage distributions. For statistical analysis, ANOVA was employed to analyze normally distributed data, while the Kruskal-Wallis H test was applied to handle non-normally distributed data. When it came to categorical data comparisons, the chi-square test or Fisher’s exact test was selected based on the data set’s suitability.

We employed multivariate logistic regression analysis to determine the independent association between AG and the risk of 28-day mortality. Addressing confounding as a critical concern in our analysis, we executed various statistical models to ascertain the robustness of our findings. Subgroup analyses were predefined according to specific subgroup variables. The presence of heterogeneity among subgroups was evaluated by incorporating an interaction term into the model, representing the interaction between the two predictor variables. To manage missing data, we employed multiple imputation with five iterations using a chained equation method, following the R mice package implementation (15). This method was selected to bolster statistical power and reduce biases that might arise due to missing data. To ensure the robustness of our findings, we conducted sensitivity analyses to determine the influence of various models of association inference on our conclusions. The effect sizes and *p*-values from these models were computed, documented, and subjected to comparison.

All analyses were conducted using R Statistical Software (Version 4.3.1) and the Free Statistics Analysis Platform (Version 2.0) (16). We considered a two-sided *P*-value of less than 0.05 to indicate statistical significance.

## Results

### Baseline demographic and clinical characteristics

A total of 489 ICU-admitted cholangitis patients were stratified into quartiles by serum anion gap (AG) levels (Q1: < 11.0; Q2:12.0–14.0; Q3:15.0–16.0; Q4: > 17.0). Baseline demographics (age, gender, race, marital status) showed no significant intergroup differences (all *p* > 0.05). However, higher AG quartiles exhibited progressively elevated incidences of acute kidney injury (AKI: Q1 = 55.8% vs. Q4 = 85.8%), sepsis (Q1 = 75.6% vs. Q4 = 92.6%), and CRRT utilization (Q1 = 3.5% vs. Q4 = 17.9%, all *p* < 0.001). Laboratory parameters reflecting inflammation (WBC), coagulation (PT, PTT, INR), and hepatorenal dysfunction (ALT, AST, bilirubin, urea nitrogen, creatinine) significantly worsened with increasing AG (all *p* < 0.001). Disease severity scores (SAPS II: 36.5 ± 13.2 in Q1 vs. 48.4 ± 16.7 in Q4; SOFA: 5.7 ± 3.3 vs. 9.1 ± 4.3) and 28-day mortality (Q1 = 8.1% vs. Q4 = 30.9%, *p* < 0.001) escalated markedly across quartiles (Table 1). These findings underscore AG as a robust biomarker for systemic inflammation, multi-organ failure, and mortality risk in critically ill cholangitis patients.

**TABLE 1:** Baseline characteristics of cholangitis according to quartiles of anion gap.

Variables	Total (*n* = 489)	Q1 (*n* = 86)	Q2 (*n* = 153)	Q3 (*n* = 88)	Q4 (*n* = 162)	*p*
Age, mean ± SD	63.2 ± 12.4	61.9 ± 12.8	63.3 ± 13.0	64.1 ± 11.4	63.4 ± 12.2	0.690
Gender, *n* (%)						0.204
Female	201 (41.1)	38 (44.2)	70 (45.8)	37 (42)	56 (34.6)	
Race, *n* (%)						0.319
Black	38 (7.8)	5 (5.8)	12 (7.8)	8 (9.1)	13 (8)	
White	342 (69.9)	63 (73.3)	106 (69.3)	59 (67)	114 (70.4)	
Asian	29 (5.9)	1 (1.2)	14 (9.2)	3 (3.4)	11 (6.8)	
Other	80 (16.4)	17 (19.8)	21 (13.7)	18 (20.5)	24 (14.8)	
Marital, *n* (%)						0.249
Single	146 (29.9)	27 (31.4)	40 (26.1)	21 (23.9)	58 (35.8)	
Married	237 (48.5)	36 (41.9)	78 (51)	47 (53.4)	76 (46.9)	
Other	106 (21.7)	23 (26.7)	35 (22.9)	20 (22.7)	28 (17.3)	
Hypertension, *n* (%)						0.410
Yes	195 (39.9)	32 (37.2)	68 (44.4)	37 (42)	58 (35.8)	
Diabetes, *n* (%)						0.296
Yes	162 (33.1)	21 (24.4)	52 (34)	31 (35.2)	58 (35.8)	
AKI, *n* (%)						<0.001
Yes	355 (72.6)	48 (55.8)	101 (66)	67 (76.1)	139 (85.8)	
Sepsis, *n* (%)						0.002
Yes	411 (84.0)	65 (75.6)	126 (82.4)	70 (79.5)	150 (92.6)	
CRRT, *n* (%)						<0.001
Yes	37 (7.6)	3 (3.5)	2 (1.3)	3 (3.4)	29 (17.9)	
Urea nitrogen, median (IQR)	20.0 (12.0, 33.0)	14.0 (10.0, 22.0)	15.0 (10.0, 24.0)	20.0 (13.8, 30.8)	32.0 (19.0, 53.8)	<0.001
Creatinine, median (IQR)	1.0 (0.7, 1.6)	0.7 (0.6, 1.1)	0.8 (0.6, 1.2)	1.1 (0.8, 1.6)	1.6 (1.0, 2.8)	<0.001
Sodium, mean ± SD	137.2 ± 5.5	137.2 ± 5.4	137.4 ± 5.0	136.9 ± 4.7	137.1 ± 6.4	0.927
Potassium, mean ± SD	4.1 ± 0.8	3.9 ± 0.6	3.9 ± 0.6	4.0 ± 0.7	4.3 ± 0.9	<0.001
Bicarbonate, mean ± SD	21.5 ± 4.5	24.1 ± 4.0	22.9 ± 3.7	21.5 ± 3.9	18.7 ± 4.4	<0.001
WBC, mean ± SD	15.2 ± 8.6	12.1 ± 7.3	13.4 ± 7.1	17.0 ± 10.2	17.5 ± 8.7	<0.001
RBC, mean ± SD	3.4 ± 0.8	3.2 ± 0.7	3.4 ± 0.7	3.5 ± 0.7	3.5 ± 0.9	0.069
PLT, mean ± SD	196.2 ± 114.2	194.5 ± 126.6	194.8 ± 108.8	207.2 ± 108.7	192.4 ± 115.8	0.795
Hemoglobin, Mean ± SD	10.3 ± 2.2	9.8 ± 2.1	10.2 ± 2.1	10.4 ± 2.1	10.5 ± 2.4	0.140
RDW, mean ± SD	16.2 ± 2.8	16.2 ± 2.7	15.7 ± 2.3	16.5 ± 3.1	16.4 ± 3.1	0.100
Hematocrit, mean ± SD	31.1 ± 6.7	29.6 ± 5.8	31.0 ± 6.4	31.1 ± 6.3	31.9 ± 7.4	0.080
PLT, mean ± SD	196.2 ± 114.2	194.5 ± 126.6	194.8 ± 108.8	207.2 ± 108.7	192.4 ± 115.8	0.795
PT, mean ± SD	19.3 ± 11.5	17.7 ± 6.5	17.2 ± 7.0	18.9 ± 10.9	22.3 ± 16.0	<0.001
PTT, mean ± SD	38.2 ± 18.0	35.6 ± 12.4	34.5 ± 13.7	40.6 ± 24.0	41.6 ± 19.4	0.002
INR, mean ± SD	1.8 ± 1.1	1.6 ± 0.6	1.6 ± 0.7	1.7 ± 1.0	2.1 ± 1.5	<0.001
ALT, median (IQR)	106.5 (46.8, 215.2)	73.5 (36.5, 134.0)	103.0 (36.0, 204.0)	113.5 (54.8, 224.8)	125.0 (65.0, 257.0)	<0.001
Age, mean ± SD	63.2 ± 12.4	61.9 ± 12.8	63.3 ± 13.0	64.1 ± 11.4	63.4 ± 12.2	0.690
AST, median (IQR)	110.5 (59.5, 242.2)	88.0 (44.0, 136.8)	101.0 (49.0, 183.0)	111.5 (62.5, 273.8)	169.0 (79.0, 350.0)	<0.001
Bilirubin total, median (IQR)	4.1 (1.9, 7.0)	2.8 (1.1, 6.1)	3.5 (1.7, 6.1)	4.3 (2.2, 6.7)	4.9 (2.7, 8.7)	<0.001
SAPS II, mean ± SD	41.4 ± 15.2	36.5 ± 13.2	36.6 ± 12.3	41.4 ± 13.8	48.4 ± 16.7	<0.001
SOFA, mean ± SD	7.2 ± 4.0	5.7 ± 3.3	5.8 ± 3.0	7.3 ± 4.1	9.1 ± 4.3	<0.001
28-day mortality, *n* (%)						<0.001
Yes	98 (20.0)	7 (8.1)	22 (14.4)	19 (21.6)	50 (30.9)	

AKI, acute kidney injury; CRRT, continuous renal replacement therapy; WBC, white blood cell; RBC, red blood cell; PLT, platelet; RDW, red cell distribution width; PT, prothrombin time; PTT, partial thromboplastin time; INR, international normalized ratio; ALT, alanine aminotransferase; AST, aspartate aminotransferase.

### Relationship between serum anion gap and mortality

**FIGURE 2:**
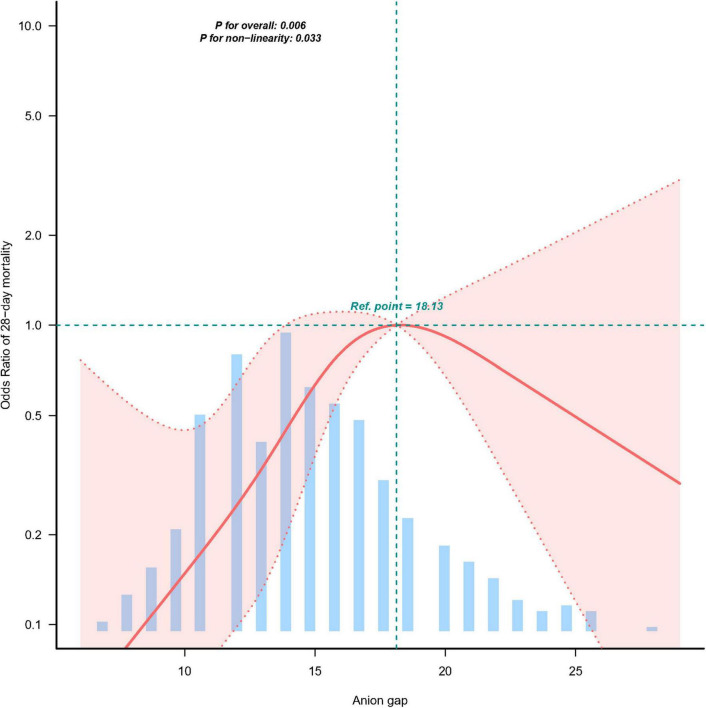
The relationship between AG and ICU 28-day mortality.

Multivariable logistic regression demonstrated a significant association between elevated serum anion gap (AG) and increased 28-day mortality (Table 2). As a continuous variable, AG was associated with a 13% increase in 28-day mortality after adjusting for all confounding factors (OR 1.13, 95% CI 1.03–1.124, *p* = 0.010). When categorized into quartiles based on AG levels, a significant increase in 28-day mortality was observed among ICU cholangitis patients as AG levels rose. Smoothed curve fitting and restricted cubic spline analysis demonstrated an inverted J-shaped relationship between AG levels and ICU 28-day mortality, with an inflection point at 18.13 mEq/L (Figure 2 and Table 3). Using segmented multivariate logistic regression models to account for the distinct slopes around this point, the likelihood ratio test yielded a *P*-value of 0.003 (Table 3). For AG levels below 18.13 mEq/L, each unit increase was associated with a 13.3% higher risk (OR 1.133, 95% CI 1.001–1.283). Above 18.13 mEq/L, no significant association was observed (OR 0.883, 95% CI 0.724–1.077). Conversely, when AG levels were above 18.13 mEq/L, each unit decrease corresponded to an 11.7% lower risk (OR 0.883, 95% CI 0.724–1.077) (Table 3).

**TABLE 2:** Association between serum AG and 28-day mortality in patients with cholangitis.

Variable	Non-adjusted model		Model I		Model II		Model III	
	OR (95% CI)	*P*-value	OR (95% CI)	*P-*value	OR (95% CI)	*P-*value	OR (95% CI)	*P-*value
AG	1.13 (1.08∼1.19)	<0.001	1.13 (1.08∼1.19)	<0.001	1.03 (0.97∼1.1)	0.349	1.13 (1.03∼1.24)	0.010
AG quartile								
Q1	1(Ref)		1(Ref)		1(Ref)		1(Ref)	
Q2	1.9 (0.77∼4.64)	0.162	1.94 (0.79∼4.8)	0.149	2.26 (0.84∼6.06)	0.105	3.9 (1.25∼12.24)	0.019
Q3	3.11 (1.23∼7.84)	0.016	3.06 (1.21∼7.77)	0.018	2.62 (0.94∼7.28)	0.066	4.79 (1.41∼16.28)	0.012
Q4	5.04 (2.17∼11.69)	<0.001	5.09 (2.17∼11.93)	<0.001	2.93 (1.11∼7.69)	0.029	7.33 (2.24∼24.05)	0.001
P for trend		<0.001		<0.001		0.046		0.002

Model I: adjust for age, gender, race, marital. Model II: model 2 + hypertension, diabetes, AKI, sepsis, CRRT, SAPS II, SOFA. Model III: adjusted for all covariates. Adjust for age, gender, race, marital, hypertension, diabetes, AKI, sepsis, CRRT, SAPS II, SOFA.

**TABLE 3:** The non-linear relationship between AG and ICU 28-day mortality.

Item	Breakpoint.OR (95%CI)	*P*-value
E_BK1	18.13 (17.847, 18.417)	NA
Slope1	1.13 (1.001∼1.283)	0.0491
Slope2	0.88 (0.724∼1.077)	0.2187
Likelihood ratio test	–	0.003
Non-linear test × 1	–	0.011
Non-linear test × 2	–	0.024

### Subgroup analysis

**FIGURE 3:**
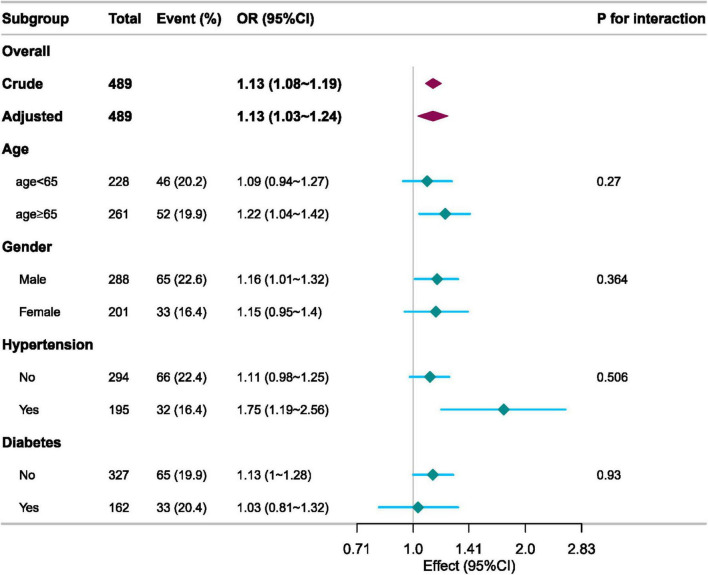
Subgroup analyses of the AG associated with ICU 28-day mortality.

We used age (<65 vs. ≥65 years), sex (male vs. female), hypertension, and diabetes as stratification variables to assess effect measures and create a forest plot (Figure 3). The study revealed statistically significant associations between elevated ICU 28-day mortality and various subgroups, including age, sex, and hypertension. The results for these groups revealed no significant interactions.

## Discussion

Acute cholangitis is a critical clinical syndrome characterized by infection of the biliary tract, often leading to severe complications such as sepsis and multi-organ failure (17). The present study explores the prognostic significance of serum anion gap (AG) in patients with acute cholangitis, focusing on its association with 28-day all-cause mortality. Utilizing a retrospective cohort design, data from the MIMIC-IV database were analyzed, encompassing a large and diverse population of ICU-admitted cholangitis patients. Our study found an OR of 1.13 for mortality associated with elevated AG in acute cholangitis, with a threshold at 18.13 mEq/L. In comparison, Zhu et al. reported an OR of 1.15 for mortality in sepsis patients with elevated AG, while Li et al. found a threshold of 16 mEq/L in ARDS patients (8, 9). The stronger association observed in our study compared to ARDS may reflect disease-specific metabolic derangements in acute cholangitis. Notably, Pan et al. developed a prediction model for mortality in ICU patients with acute cholangitis and found that AG was not included as a predictor (18). This discrepancy may be due to differences in study design, variable selection criteria, and the specific objectives of prediction models versus associative cohort studies. Our findings reveal that elevated AG levels correlate significantly with increased mortality rates, suggesting that AG may serve as a valuable biomarker in stratifying risk and guiding clinical management. This research not only highlights the relevance of AG in the context of systemic inflammation and organ dysfunction but also emphasizes the necessity for further investigation into its potential as a therapeutic target in critically ill cholangitis patients.

The implications of these results for clinical practice are substantial. Elevated AG levels may serve as a valuable tool in guiding treatment decisions and monitoring strategies for patients diagnosed with acute cholangitis. A retrospective cohort study showed a positive correlation between serum anion gap levels and all-cause mortality in unscreened adult patients, with higher serum anion gap levels associated with higher patient mortality (19). The underlying mechanisms may be rooted in the association between AG elevation and metabolic disturbances (20, 21). These disturbances encompass lactic acidosis secondary to sepsis or tissue hypoperfusion, metabolic acidosis resulting from bile acid accumulation due to biliary obstruction, and the exacerbation of AG elevation by renal tubular dysfunction in AKI (6). Additionally, the predictive value of AG for complications has been supported by studies indicating that each unit increase in AG correlates with a significant rise in the risk of adverse outcomes (22, 23). This correlation emphasizes the importance of monitoring AG levels in clinical practice to identify patients at greater risk for prolonged hospitalization and complications, thereby facilitating timely interventions. Additionally, when combined with other biochemical markers such as liver function tests and inflammatory markers, the AG can enhance the accuracy of the differential diagnosis, ultimately leading to more tailored and effective management strategies for patients (24, 25).

However, it is essential to acknowledge the limitations inherent in this study. Notably, this study did not consider albumin-corrected AG, which may be affected by hypoalbuminemia common in critically ill patients. Future studies could evaluate albumin-corrected AG to account for hypoalbuminemia in cholangitis. The retrospective design may introduce biases that affect the generalizability of our findings. Additionally, reliance on a single database, the MIMIC-IV, could limit the applicability of the results to broader populations. In our study, we identified a significant association between AG levels and risk below the 18.13 mEq/L threshold. However, the lack of significance above this threshold could stem from the limitations of our statistical model or the diverse clinical presentations of our patient cohort. Given these considerations, it would be wise to validate the clinical utility of this threshold through further prospective studies. Future research should aim to address these limitations by replicating our findings in diverse clinical settings and exploring the potential for AG-targeted interventions to improve patient outcomes. Thus, this study lays a foundation for further investigation into the role of AG in acute cholangitis, with the potential to inform clinical guidelines and enhance patient care strategies.

## Conclusion

In conclusion, the findings of this study underscore the significant association between serum anion gap and 28-day mortality in patients with acute cholangitis. Elevated AG levels emerged as a critical prognostic marker, indicating a heightened risk of mortality and multi-organ failure in this patient population. These insights provide a valuable foundation for improving clinical decision-making and patient management strategies in critical care settings. Future research should focus on validating these results in diverse cohorts and exploring potential therapeutic interventions aimed at modulating AG levels to enhance patient outcomes.

## Data Availability

The original contributions presented in this study are included in this article/Supplementary material, further inquiries can be directed to the corresponding author.
