# Supplementary material for: Anion gap associated with 28-days all-cause mortality in Acute cholangitis patients admitted to the intensive care unit in MIMIC-IV database: a retrospective cohort study
Source: Front Med (Lausanne). 2025 May 23;12:1591096. doi: 10.3389/fmed.2025.1591096 (PMC12142621; doi:10.3389/fmed.2025.1591096)
Supplement: Supplementary file 1 [file Table_1.docx]

**Supplementary Table 1** Univariate and Multivariate analysis.

| Variable | crude.OR (95%CI) | crude.P value | adj.OR  (95%CI) | adj.P value |
| --- | --- | --- | --- | --- |
| Anion gap | 1.13 (1.08~1.19) | <0.001 | 1.13 (1.03~1.24) | 0.01 |
| Age | 1.01 (0.99~1.03) | 0.328 | 1.01 (0.98~1.05) | 0.467 |
| Gender |  |  |  |  |
| Male | 1(Ref) |  |  |  |
| Female | 0.67 (0.42~1.07) | 0.096 | 0.61 (0.3~1.22) | 0.162 |
| Race |  |  |  |  |
| Black | 1(Ref) |  |  |  |
| White | 1.28 (0.51~3.18) | 0.601 | 2.12 (0.46~9.72) | 0.334 |
| Asian | 1.39 (0.4~4.87) | 0.605 | 2.68 (0.37~19.47) | 0.331 |
| Other | 1.78 (0.65~4.87) | 0.263 | 3.56 (0.7~18.01) | 0.125 |
| Marital |  |  |  |  |
| Single | 1(Ref) |  |  |  |
| Married | 1.18 (0.7~1.99) | 0.537 | 0.99 (0.45~2.16) | 0.978 |
| Other | 1.09 (0.58~2.05) | 0.793 | 1.11 (0.44~2.8) | 0.828 |
| AKI |  |  |  |  |
| No | 1(Ref) |  |  |  |
| Yes | 2.93 (1.57~5.46) | 0.001 | 1.17 (0.48~2.82) | 0.731 |
| Sepsis |  |  |  |  |
| No | 1(Ref) |  |  |  |
| Yes | 1.3 (0.69~2.48) | 0.418 | 0.51 (0.2~1.31) | 0.163 |
| CRRT |  |  |  |  |
| No | 1(Ref) |  |  |  |
| Yes | 3.44 (1.72~6.87) | <0.001 | 0.68 (0.22~2.05) | 0.489 |
| SAPSⅡ | 1.07 (1.06~1.09) | <0.001 | 1.09 (1.05~1.13) | <0.001 |
| SOFA | 1.21 (1.14~1.28) | <0.001 | 0.98 (0.85~1.13) | 0.783 |
| Hypertension |  |  |  |  |
| No |  |  |  |  |
| Yes | 0.68 (0.42~1.08) | 0.104 | 0.89 (0.46~1.73) | 0.738 |
| Diabetes |  |  |  |  |
| No |  |  |  |  |
| Yes | 1.03 (0.65~1.65) | 0.898 | 0.9 (0.46~1.78) | 0.771 |
| WBC | 1.01 (0.99~1.04) | 0.267 | 0.99 (0.95~1.02) | 0.485 |
| RBC | 0.54 (0.39~0.75) | <0.001 | 0.54 (0.17~1.73) | 0.302 |
| PLT | 1 (1~1) | 0.519 | 1 (0.99~1) | 0.113 |
| Hemoglobin | 0.83 (0.74~0.92) | 0.001 | 0.77 (0.38~1.57) | 0.474 |
| RDW | 1.34 (1.23~1.45) | <0.001 | 1.26 (1.11~1.43) | <0.001 |
| Hematocrit | 0.94 (0.9~0.97) | 0.001 | 1.13 (0.88~1.46) | 0.325 |
| Sodium | 0.94 (0.9~0.97) | 0.001 | 0.97 (0.91~1.02) | 0.214 |
| Potassium | 1.56 (1.18~2.06) | 0.002 | 1.37 (0.89~2.12) | 0.152 |
| PT | 1.02 (1~1.04) | 0.032 | 1.65 (1.01~2.7) | 0.045 |
| PTT | 1.02 (1.01~1.03) | <0.001 | 1.01 (0.99~1.03) | 0.175 |
| INR | 1.2 (1~1.43) | 0.044 | 0 (0~0.84) | 0.043 |
| ALT | 1.09 (1.06~1.13) | <0.001 | 1.05 (1~1.1) | 0.067 |
| AST | 1 (1~1) | 0.006 | 0.99 (0.99~1) | <0.001 |
| Bilirubintotal | 1 (1~1) | 0.925 | 1 (1~1.01) | 0.001 |
| Ureanitrogen | 1.02 (1.01~1.02) | <0.001 | 0.99 (0.98~1.01) | 0.591 |
| Creatinine | 1.19 (1.06~1.34) | 0.003 | 0.74 (0.54~1.02) | 0.064 |
