# Supplementary figures and images for: Anion gap associated with 28-days all-cause mortality in Acute cholangitis patients admitted to the intensive care unit in MIMIC-IV database: a retrospective cohort study
Source: Front Med (Lausanne). 2025 May 23;12:1591096. doi: 10.3389/fmed.2025.1591096 (PMC12142621; doi:10.3389/fmed.2025.1591096)

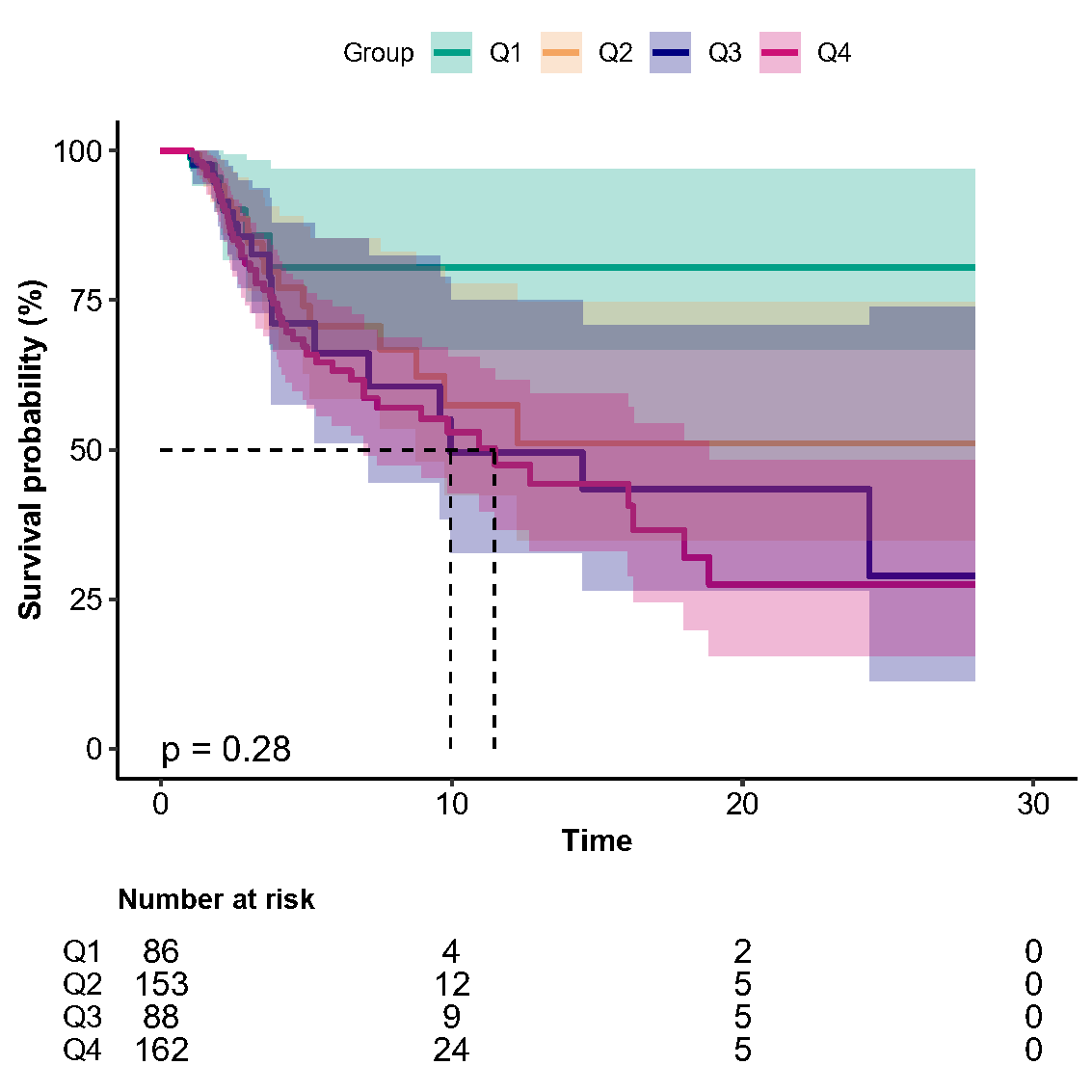

Supplement: Supplementary file 2 [file Image_1.tiff]
